# Troublesome Tinnitus in Children: Epidemiology, Audiological Profile, and Preliminary Results of Treatment

**DOI:** 10.1155/2012/945356

**Published:** 2011-07-19

**Authors:** G. Bartnik, A. Stępień, D. Raj-Koziak, A. Fabijańska, I. Niedziałek, H. Skarżyński

**Affiliations:** Tinnitus Clinic, Institute of Physiology and Pathology of Hearing, AK Kampinos 1, 01-943 Warsaw, Poland

## Abstract

*Introduction*. Although tinnitus often has a significant impact on individual's life, there are still few reports relating to tinnitus in children. In our tinnitus clinic, children with distressing tinnitus constitute about 0,5% of all our patients. *Objectives*. The aim of this study was to analyse children with troublesome tinnitus as regards epidemiology, audiological profile, and preliminary effects of the therapy. *Methods*. A retrospective study was carried out involving the cases of 143 children consulted in our Tinnitus Clinic in 2009. The selected group with troublesome tinnitus was evaluated and classified for proper category of Tinnitus Retraining Therapy (TRT). *Results*. The study showed that 41.3% of the children suffered from bothersome tinnitus. In this group 44.1% of the patients demonstrated normal hearing. The success of the therapy after 6 months was estimated on 81.4% of significant improvement. *Conclusions*. It is recommended that a questionnaire include an inquiry about the presence of tinnitus during hearing screening tests.

## 1. Introduction

Tinnitus is perception of sound like buzzing, ringing, roaring, clicking, pulsations, and other noises in the ear, ears, or in the head occurring without an outside acoustic stimulus. Tinnitus is a symptom, not a disease, and as such can arise as a result of inappropriate activity at any point in the auditory pathway. The neural plasticity, responsible for tinnitus, appears as an adaptation or compensation, at a central nervous system level, following peripheral damage. Tinnitus may be caused by a variety of pathologies, illnesses, medications, allergies, dietary changes, stresses, or traumatic events. It is often caused by pathologies related to the ear, head, and neck area like: head trauma or whiplash. One of the most common causes of tinnitus involves noise exposure. It can be both: long term and produced by brief loud sound. Long-term exposure to noise or acoustic trauma can produce substantial and often irreparable damage to the delicate structures of the inner ear—especially outer hair cells. In the majority of cases, tinnitus is clearly related to some changes in the inner ear. At the same time tinnitus is not necessarily associated with hearing loss. About 40% of tinnitus patients have also the symptoms of decreased loudness tolerance called hyperacusis [1].

The prevalence of tinnitus increases with age. In older people, tinnitus occurs most often in conjunction with the hearing problems connected with ageing or it can be associated with general diseases. About 5% of adult Poles suffer from constant tinnitus [2]. Research into the prevalence of tinnitus in a pediatric population in Poland on large scale has been continued by the Institute of Physiology and Pathology of Hearing for last years. The initial results show that 12.8% of seven-year-old children have tinnitus in our country [3]. There are few epidemiological studies on the prevalence of tinnitus in children in the literature. In a group of children with normal hearing, the prevalence of tinnitus has been reported to be between 6% and 36%. In a study by Nodar [4], there was 15% of those patients. Other researchers, Mills et al. [5] estimated that 29% of normally hearing children complain about tinnitus, and only 9,6% reported the troublesome tinnitus [5]. The latest paper by Holgers [6] highlights about 13% of pediatric patients with normal hearing and tinnitus [6]. In children with hearing loss, the prevalence of tinnitus is reported to be as high as 55% [7]. Graham and Butler [8] found the prevalence to be twice as high (66%) in children with mild to moderate hearing impairment than in those with severe to profound hearing loss (29%) [8]. Mills and Cherry [9] estimated 43,9% of children with conductive hearing loss and tinnitus compared to 29,5% of those with sensorineural hearing impairment [9]. 

However, tinnitus is a common disorder in childhood, and only 3% to 6.5% of young patients spontaneously complain about it [7, 10]. In a survey showing the children with that symptom presented to audiologists, Martin and Snashall reported that 83% of children suffered from bothersome tinnitus [11]. Overall, children with normal hearing found tinnitus more troublesome and presented higher levels of anxiety than those with hearing loss [12]. Tinnitus primarily produces psychological distress. Even if a child does not report the existence of tinnitus, it may cause serious consequences such as difficulties in concentration, stress, fatigue, irritation, sleep disturbances, deterioration in learning, poor attentiveness, and emotional distress. In older children, complaints relating to sleep disturbance, auditory interference, and emotional distress have been consistently found in subsequent studies [13]. On the basis of our experience, even though some children really suffer from tinnitus, they usually do not need an additional psychological help. 

A treatment derived from the neurophysiological model of tinnitus applied on time for young patient is a good and effective therapeutic tool. It is a specific form of sound therapy and counseling [1] called Tinnitus Retraining Therapy (TRT) [1]. This management facilitates habituation to tinnitus by suppressing negative reactions and associations caused by tinnitus as well as suppressing or even eliminating its perception. On the basis of the interview and some of the audiological tests, the patients are divided into five categories of treatment. The factors that determine ration to appropriate category are the impact of tinnitus on the patient's life, presence or absence of hyperacusis, subjective hearing loss, the presence of prolonged worsening of tinnitus, and/or hyperacusis after exposure to loud sound (Table 1). 

The therapy needs at least 18 months of training to have stable effects. A positive result of the therapy is achieved by about 80% of adult patients and approximately 90% of children after 18 months of treatment [14, 15].

## 2. Materials and Methods

This study considered 143 children with tinnitus aged under eighteen who were consulted in our clinic in 2009. All of them were accompanied by at least one parent for the initial assessment, where detailed information about a child's life, tinnitus onset, and its influence upon life was gathered. 

The children underwent the following protocol: case history, filling in the initial contact questionnaire, audiological evaluation, medical evaluation, diagnosis, and selection for the treatment category. The hearing screening tests were performed using: pure tone audiometry with air conduction in the range of frequency 0,25–8 kHz and impedance audiometry with acoustic reflexes. Children with the abnormal tympanometry test and those whose the only problem was hyperacusis were excluded. The degree of hearing loss, when presented, was classified according to BIAP (20–40 dB: mild, 40–70 dB: moderate, 70–90 dB: severe, >90 dB: profound).

To report tinnitus as troublesome, there were three parameters displayed on the visual scale from 0 to 10 with 10 being the worst. The children with parents help subjectively evaluated on this scale the degree of annoyance caused by tinnitus, the impact on their activities, and the intensity of tinnitus. Only those who indicated five or more were included in our study. 

All the children with troublesome tinnitus took part in the TRT. There were no other preselection criteria except the requirements for children to be treated for at least 6 months and each of them had to be actively present at all follow-up visits. They underwent directive counseling, selection of the treatment category, fitting of the most suitable hearing aids or noise generators, follow-up counseling according to the individual needs of the patient, and an established timetable.

Treatment effects were estimated after a 6-month therapy involving filling a special questionnaire and using the visual analog scale from 0 to 10 with 10 being the worst. It comprised the parameters, which were presented in the form of questions to be answered by the child before, during, and after 6 months (Table 2).

The data acquired from the questionnaire results of the therapy were evaluated by means of two criteria: significant improvement and no improvement or deterioration. The criterion of significant improvement was the decrease of at least three of the above-mentioned parameters by a minimum of 20% and the liberation of at least one of the everyday life activities previously impaired by tinnitus.

## 3. Results

**Figure 1:**
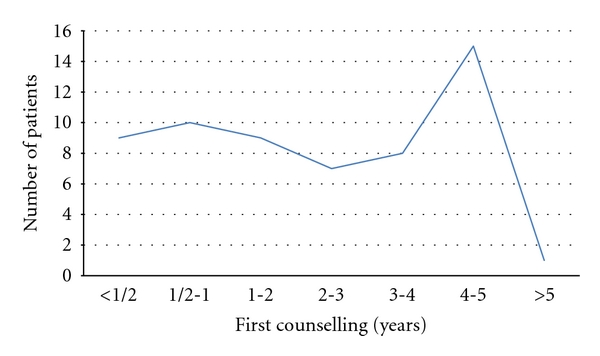
Time from the onset of tinnitus to first counseling.

**Figure 2:**
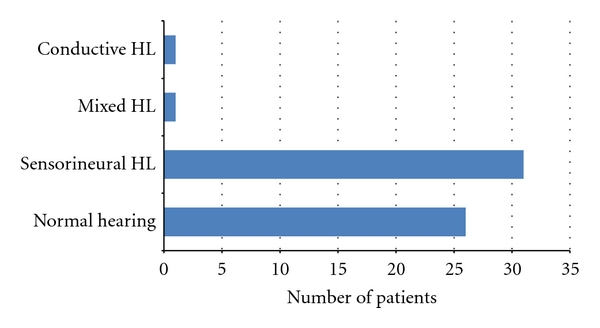
Hearing level in the study group. Hearing loss (HL).

**Figure 3:**
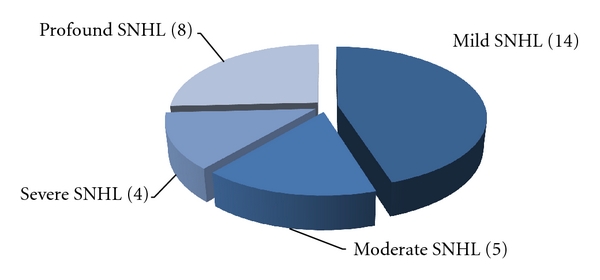
Number of children with tinnitus and different degrees of severity of sensorineural hearing loss (SNHL).

**Figure 4:**
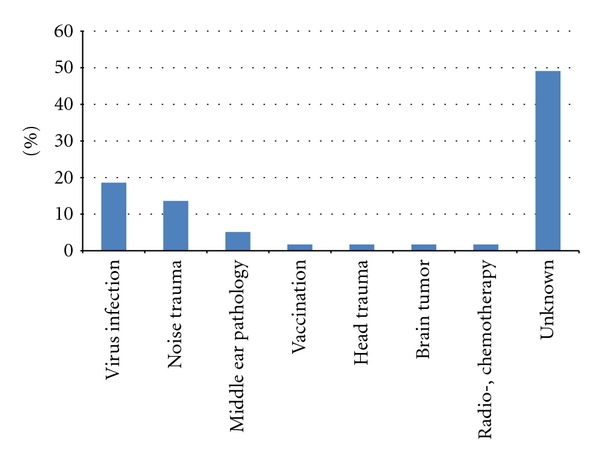
The suspected etiology of tinnitus in the study group.

Troublesome tinnitus was present in 59 children (41,3%), 31 girls (52,5%) and 28 boys (47,5%), at the average age of 14,4 (min. 7, max. 17). Some of them complained about hyperacusis (*n* = 22, 37,3%). There were significantly more girls with this symptom (*n* = 14, 63,6%) than boys (*n* = 8, 36,4%). Despite bothersome tinnitus, only 19 children (32,3%) had first audiological counseling within a year after the onset of tinnitus (Figure 1). In all, 44,1% of children (*n* = 26) demonstrated normal hearing while 55,9% (*n* = 33) had hearing impairment (Figures 2 and 3). According to the suspected etiology of tinnitus, the most common one was a clinical history of a virus infection (18,6%), and in 49,1% of cases etiology was unknown. In Figure 4, there is a detailed summary of the causes percentage distribution.

As far as the specific characteristics of tinnitus were concerned, 56 children described their tinnitus as continuous in 20 cases (33,9%), as intermittent in 36 cases (61%). There were 3 children that did not specify duration of tinnitus. The pitch of tinnitus was low (<2 kHz) in 14 children (23,7%), high (>4 kHz) in 40 children (67,8%), pulsating in 1 case (1,7%), and undefined in 3 cases (5,1%). 

**Figure 5:**
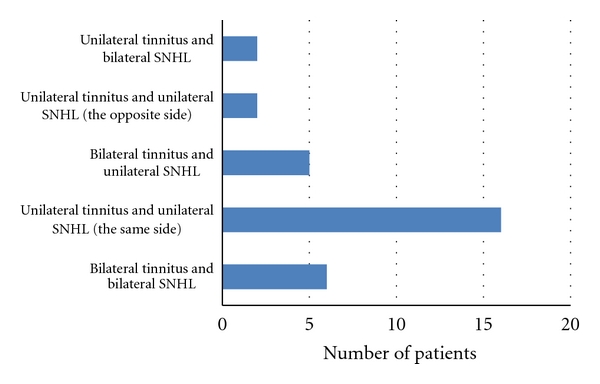
Number of children with sensorineural hearing loss (SNHL) divided into 5 groups according to laterality of tinnitus and SNHL.

Tinnitus was declared as bilateral in 29 cases (49,1%), unilateral in 28 cases (47,5%), and in the head in 2 cases (3,4%). The patients with sensorineural HL declared that unilateral tinnitus is the most common (Figure 5). It was observed that in a group of children with bilateral tinnitus, more than half had normal hearing (*n* = 18, 62, 1%).

All children with troublesome tinnitus fell into appropriate categories of the TRT: 14 children with tinnitus and normal hearing fell into category I, 32 children with tinnitus and HL belonged in category II. In category III, there were 13 children classified with tinnitus and hyperacusis as a main problem; 12 of them had normal hearing. 

Enriched environmental sounds and silence avoidance were recommended to all patients. The children in category I were advised to use noise generators, in category II to use hearing aid and in category III to use noise generators behind ears. The children with conductive and mixed HL underwent myringotomy with tube insertion additionally. However, less than half of the patients ( 47,5%) strictly observed the orders.  Table 3 shows how many children in each category used recommended devices.

The preliminary results were evaluated after 6 months of therapy. Management success: significant improvement was observed in 48 cases (81,4%). There was no change in perception of tinnitus in 9 cases (15,2%). In 2 cases (3,4%), the effect of the therapy was undefined. Results of the TRT in subsequent categories are presented in Table 4.

The TRT has been continued by about 30% of children. Characteristics of this group after 1 year of treatment will be shown in the next study.

## 4. Discussion

This small-scale preliminary study showed, in contrast with the data by Martin and Snashall, that less than half of the the patients with tinnitus reported that the ailment was bothering them [11]. Over 50% of children with tinnitus seen in our clinic had hearing impairment. Similar results have been presented by Holgers [16] who estimated a prevalence of tinnitus in children between 23 and 64% [16].

A past medical history of middle-ear pathology in a pediatric group with tinnitus was performed in 35,6% of cases and did not seem to be a significant factor in the pathogenesis of children's tinnitus. Similar results were presented by Mills and Cherry, Martin and Snashall, and Viani [9, 11, 17]. Those researchers found no statistical difference between the group without middle ear pathology and the group who had positive history of middle ear disorder.

Although tinnitus seems to be a serious problem for over 40% of children, causing many disturbances in their lives, only about one-third of them decides to get audiological help. 

After just 6 months of treatment, most of the children with troublesome tinnitus gained benefits from the therapy. What is more, for over 80% of pediatric population, the TRT is limited to sound and noise generators.

About half of the children in our study had normal hearing. It indicated the greater significance of other factors than hearing impairment. Similar results were obtained by Graham and Butler [8].

## 5. Conclusions

Troublesome tinnitus is a quite frequent complaint among children and concerns both normal hearing children as well as those with some degree of hearing loss. This problem still needs careful attention and research. Until we gain more knowledge on the development of bothersome tinnitus in pediatric population, audiologists have to rely on the reports based on adults. It is recommended that an inquiry about the presence of tinnitus during hearing screening tests at school should be included in the questionnaire. Treatment strategies should be applied individually and involve both the parents and children.

## Figures and Tables

**Table 1:** Categories of TRT mixing point-level close to the place where the sound from the instrument blends with the tinnitus.

Category	0	I	II	III	IV
Tinnitus	Small problem	Serious problem	Serious problem	Present/absent	Present/absent

Hyperacusis	Absent	Absent	Absent	Present	Present/absent

Noise exposure	No prolonged effect	No prolonged effect	No prolonged effect	No prolonged effect	Prolonged effect

Subjective Hl	Absent	Absent	Significant	Irrelevant/ significant	Irrelevant/significant

Main problem	Tinnitus	Tinnitus	HL	Hyperacusis	Tinnitus or hyperacusis

Treatment	Avoid silence recommendation	Noise generators set at mixing point	Hearing aids with/without noise generators set below mixing point	Noise generator firstly set at hearing threshold then gradually increased to mixing point	Noise generators firstly set at hearing threshold then gradually increased to mixing point

**Table 2:** The questionnaire that was used to estimate results of treatment.

Measure the following parameters using visual analog scale (VAS):	VAS (10 means the worst)
(1) The impact of tinnitus with/without hyperacusis on various everyday activites	0-1-2-3-4-5-6-7-8-9-10

(2) Percentage of time of being aware of tinnitus	0-1-2-3-4-5-6-7-8-9-10

(3) The degree of annoyance	0-1-2-3-4-5-6-7-8-9-10

(4) The impact of tinnitus on your life	0-1-2-3-4-5-6-7-8-9-10

(5) The intensity of tinnitus	0-1-2-3-4-5-6-7-8-9-10

(6) The level of distress caused by tinnitus	0-1-2-3-4-5-6-7-8-9-10

**Table 3:** The number of children in each category of TRT who used recommended devices.

Category of TRT	Number of children	Devices used
I	14	13 bed-side noise generators

II	32	12 hearing aids, 4 bed-side noise generators

III	13	3 noise generators behind ears, 7 bed-side noise generators.

**Table 4:** The result of TRT in the subsequent categories.

Category of TRT	Significant improvement	No improvement	Undefined result
I (14 children)	12	1	1

II (32 children)	28	4	0

III (13 children)	8	4	1
